# Robotic assisted CyberKnife radiosurgery for the treatment of iris melanoma

**DOI:** 10.1038/s41598-021-84290-x

**Published:** 2021-03-11

**Authors:** Valerie Schmelter, Sarah Heidorn, Alexander Muacevic, Siegfried G. Priglinger, Paul Foerster, Raffael Liegl

**Affiliations:** 1 Department of Ophthalmology, Ludwig-Maximilians University Munich, Mathildenstr. 8, 80336 Munich, Germany; 2 European CyberKnife Center Munich, Munich, Germany

**Keywords:** Cancer, Eye cancer

## Abstract

Iris melanoma is a rare form of uveal melanoma with potential metastic spread. Treatment options include surgical resection, enucleation or irradiation. We analysed visual outcome, complication appearance and management in eight patients with iris melanoma following robotic-assisted CyberKnife treatment. Consecutive patients from the Department of Ophthalmology at University of Munich were included in the study if they had an iris melanoma that was treated with CyberKnife and had a minimum follow-up of 12 months. We evaluated tumor thickness, largest diameter, visual acuity and complications. 8 patients were included in this report. The median age was 74 years (range: 53–86 years). The median follow-up was 23 months (range 12–48 months). Tumor thickness decreased from 2.1 to 1.4 mm on average. Four out of eight patients showed stable or increased visual acuity compared to visual acuity at first visit. We did not find a correlation of applied radiation volume or radiation dose on visual outcome. Radiation keratopathy was the most common complication in five patients. No recurrences were noted. Robotic-assisted radiosurgery following CyberKnife is a promising non-invasive, single session treatment option for iris melanoma with comparable results regarding recurrence rate or complications to brachytherapy and proton beam therapy. All included patients showed good visual outcome.

## Introduction

Uveal melanoma is the most frequent intraocular malignancy and can be subcategorized into uveal melanoma developing in the choroid, in the ciliary body, in the iris or a combination of any of these locations. The vast majority of uveal melanoma is found in the choroid (80–90%), whereas ciliary body and particularly iris affection is considerably less common with approximately 10% and 4% respectively in the mid-aged to older population. Younger patients, although less commonly affected by uveal melanoma in general, are more frequently affected by iris melanoma with around 20% of all uveal melanoma^1^.

Several factors have been established that are linked to a higher risk of developing iris melanoma. As with other uveal melanoma, fair skin, light eye color as well as cutaneous nevi, particularly when atypical, are risk factors for developing iris melanoma^2^.

The presence of an iris nevus is quite common, representing 25% of all iris lesions in children and 47% of all iris lesions in middle-aged and senior adults^3^. It is also a risk factor for the later development of an iris melanoma. The rate of transformation of iris nevus into melanoma is controversial and has been estimated at nearly 5% after 5 years^4^; higher rates have also been reported with some diagnostic challenges^5^. Several studies found predictive clinical factors for growth of iris nevus into melanoma. These clinical features include hyphema, 4:00 to 9:00 clock hour tumor location, patient age under 40 years, ectropium uveae, the presence of a feeder vessel, nodule formation and diffuse malignancy^6,7^.

In general, Iris melanomas demonstrate low metastatic potential compared to other uveal melanomas and is believed to be around 3% after 5 years and 5% after 10 years^8^. Tumor related death occurs in approximately 5–10% of patients, and increases with tumor thickness of more than 4 mm^9^.

The most appropriate form of treatment is still topic of an ongoing debate, yet radiation therapy has constantly supplanted surgical resection of the tumor lesion and particularly removal of the whole eye^10^.

Surgical resection of iris melanoma can be limited to an iridectomy or incorporate the removal of large parts of the iris including parts of the ciliary body. Sometimes this procedure needs additional radiotherapy and recurrences are often described, even years later^11,12^.

Today, most cases are either managed by teletherapy using proton or photon beam radiotherapy^13^ or plaque radiotherapy^14^.

We employed photon beam radiotherapy facilitated through the use of a linear accelerator mounted on a robotic arm, the CyberKnife system, to treat patients with iris melanoma. We report our results on eight patients that have been treated between 2014 and 2018. We analyzed visual outcome, complications including development of cataract and neovascular glaucoma, recurrences as well as overall survival.

To our knowledge, this is the first study evaluating overall outcome of CyberKnife therapy in the management of iris melanoma.

## Methods

We did a retrospective review of all patients that were diagnosed with iris melanoma and were treated with robotic assisted radiosurgery (CyberKnife, Accuray Inc., Sunnyvale, CA, USA) at the Department of Ophthalmology of the Ludwig-Maximilians-University in Munich, Germany in cooperation with the European CyberKnife Center in Munich, Germany. The study is approved by the ethics committee´s review board of the medical faculty at the Ludwig-Maximilians-University (“Ethikkomission der LMU”) for this medical records review. The study was in accordance with the Declaration of Helsinki. A minimum follow-up of 12 months was necessary to be included in this study.

We recorded age, gender, laterality, progression of visual acuity (BCVA), tumor thickness and largest diameter using ultrasound-biomicroscopy (UBM) at first visit and follow-up visits. We also recorded central retinal thickness (CRT) measured via optical coherence tomography (OCT) at each visit. We documented complications, including cataract progression, glaucoma development, radiation keratopathy, radiation retinopathy and recurrence rate as well as development of metastases and overall survival. All tumors were categorized following the updated American Joint Committee on Cancer (AJCC) classification in its eighth edition^15^. A correlation of visual acuity development with different variables, such as radiation dose on fovea, lens and optic disc as well as with total radiation volume was calculated.

Tumor recurrence was defined as any degree of documented tumor growth (in thickness or base) appreciated by ophthalmoscopy, photographic comparison with earlier visits and ultrasound biomicroscopy (UBM).

Written informed consent was obtained before treatment and risks and chances as well as treatment options (e.g. brachytherapy and proton beam therapy) were discussed with the patient. CyberKnife radiotherapy was performed as a standardized outpatient procedure as described previously^16^. In brief, standard retrobulbar anesthesia was performed to achieve akinesia of the globe within the orbit. Target volume was defined by an interdisciplinary team composed of ophthalmologists with special knowledge in the treatment of uveal melnaoma, medical physicists and radiation oncologists using gadolinium-contrast-enhanced MRI, computer tomography (CT) (1.0 and 1.2 mm slices) as well as all previously obtained clinical data including clinical examination and ultrasonography as well as ultrasound biomicroscopy results. A non-isocentric inverse algorithm was used in cooperation with a medical physicist for treatment planning (Multiplan, Accuray Incorporated, Sunnyvale, California, USA). In all but one case a doughnut shaped pattern incorporating the whole iris was developed and radiation was delivered according to this plan in a single fraction with a CyberKnife system in a net radiation time of approximately 20 min.

Data was collected and analyzed in Excel (Microsoft Corporation, Redmond, WA, USA). Statistical analysis was performed in Graphpad Prism 8.0 (Graphpad, San Diego, CA, USA).

Changes in apical tumor height and largest basal diameter were calculated with a Wilcoxon matched pairs signed rank test. Correlations were calculated using Spearman´s rho. The significance threshold was set at 0.05.

Visual acuity is displayed as logMAR with averages calculated as mean. Light perception is recorded as 2.70 logMAR.

### Ethics statement

A waiver of informed consent was granted and approved by the ethics committee´s review board of the medical faculty at the Ludwig-Maximilians-University (“Ethikkomission der LMU”) for this medical records review. The study was in accordance with the Declaration of Helsinki.

## Results

A total of 13 patients were treated between 2014 and 2018 in our department. 8 of these patients fulfilled the inclusion criteria and are described in this report. The median age was 74 years (range: 53–86 years; mean: 71 years). The median follow-up was 23 months [range 12–48 months] and the mean follow-up 26.75 months [SD ± 12.3 months].

**Figure 1:**
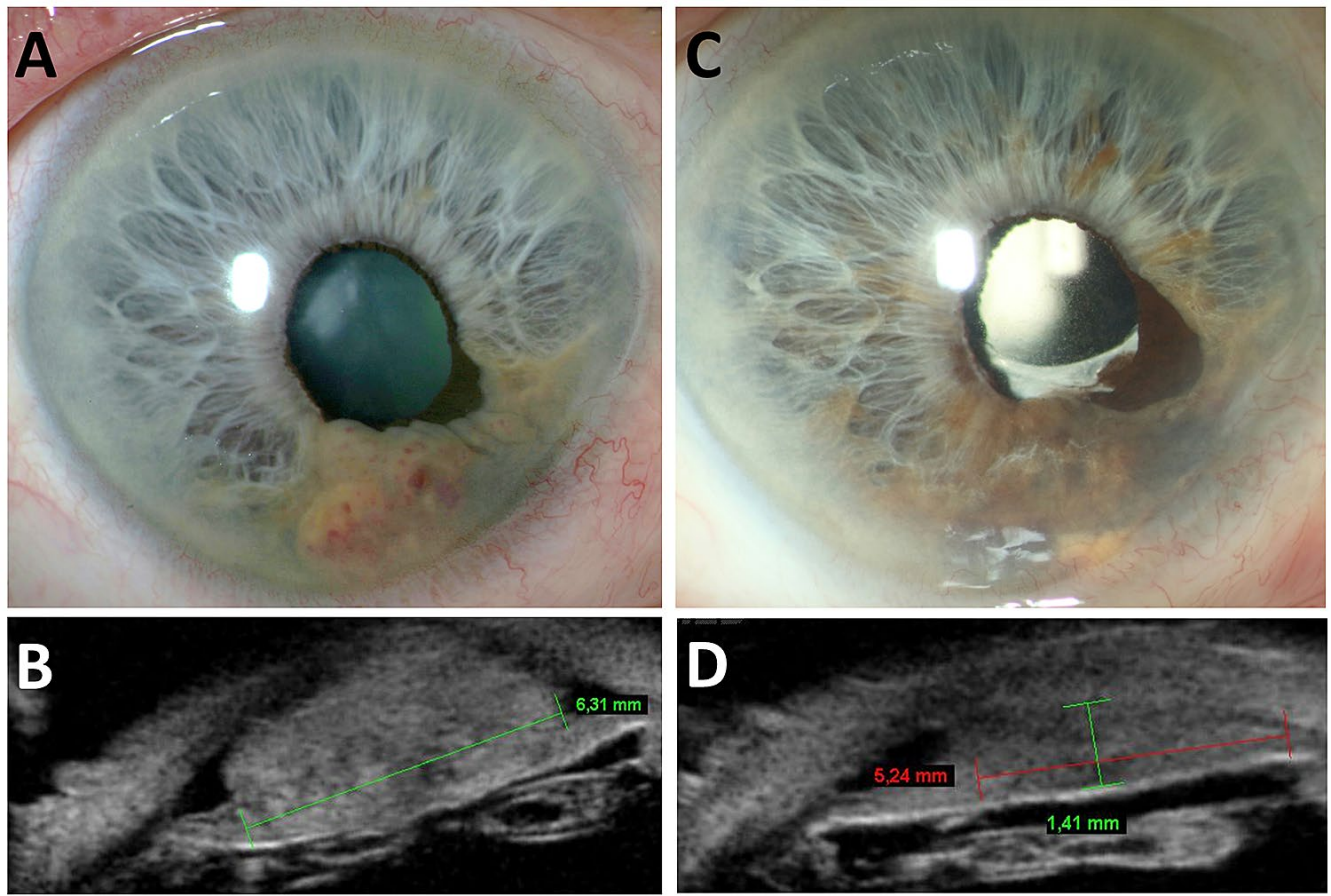
An 85 year old male patient with suspect iris lesion was observed over seven years. When enlargement of the lesion was suspected, a biopsy was performed, which eventually confirmed the diagnosis of iris melanoma. (**A**,**B**) The patient was subsequently treated with CyberKnife and responded with regression of tumor at last follow-up two years later (**C**,**D**).

Seven patients received CyberKnife treatment as first treatment option for their iris melanoma. One patient had a partial iridectomy initially, but showed signs of insufficient resection of the tumor which made a second treatment approach necessary. CyberKnife was done four months later in the earlier described algorithm. Six patients were treated solely on clinical signs that allowed for a clear diagnosis of iris melanoma. One patient had an iris biopsy beforehand, which assured the suspicion of an iris melanoma. This case is depicted in Fig. 1. The aforementioned patient who was resected as the primary mode of treatment had also an iris melanoma confirmed by histopathology of the removed specimen.

The overall follow-up time between radiation and last follow-up visit was 27 months (range: 12–48 months). (Table 1).

**Table 1:** Patient characteristics.

Pat. no	Age	Sex (female/male)	Laterality (RE/LE)	Tumor thickness FD [mm]	Tumor diameter FD [mm]	Tumor thickness FU [mm]	Tumor diameter FU [mm]	Ciliary body involvement (yes/no)	Total follow-up [months]
1	79	Male	LE	1.7	6.0	Block excision	Block excision	Yes	43
2	65	Male	LE	2.1	4.6	1.5	4.1	No	48
3	79	Female	RE	2.2	5.0	1.32	4.1	No	25
4	79	Female	LE	1.5	2.9	1.2	2.8	No	21
5	53	Male	LE	1.0	1.9	0.5	3.0	No	22
6	69	Male	RE	3.0	8.3	2.5	6.8	No	19
7	86	Male	RE	3.6	6.5	1.5	5.3	No	24
8	61	Male	RE	2.0	4.9	1.3	4.9	No	12

*FD* first diagnosis, *FU* follow-up, *RE* right eye, *LE* = left eye.

### Visual Acuity (BCVA)

Best-corrected visual acuity (BCVA) before CyberKnife treatment was 0.30 logMAR (range: 0.70–0.00 logMAR) on average. It decreased to 0.50 logMAR (range: 1.30–0.00 logMAR) one month after CyberKnife treatment. The main reason for this decrease was development of radiation keratopathy.

One year after radiotherapy five out of eight patients (62.5%) showed stable or even increased visual acuity compared to visual acuity at first visit (mean: 0.70 logMAR; range: 0.00–2.70 logMAR).

**Figure 2:**
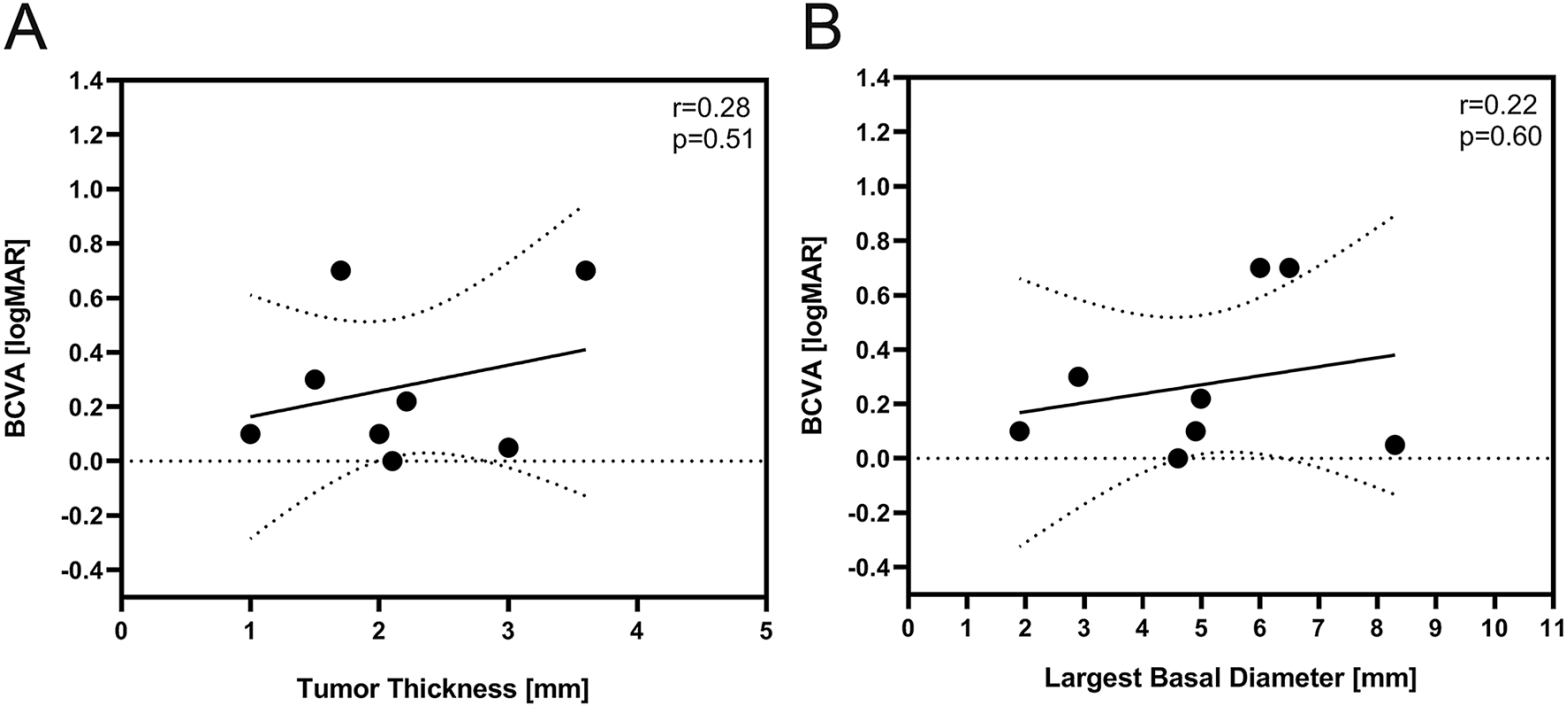
Although there was a trend for lower best corrected visual acuity [BCVA] over the course of follow-up with both increased tumor height and larger basal diameter, this was statistically not significant.

There was no statistically significant relationship between tumor thickness or largest basal diameter with initial visual acuity (Fig. 2).

**Figure 3:**
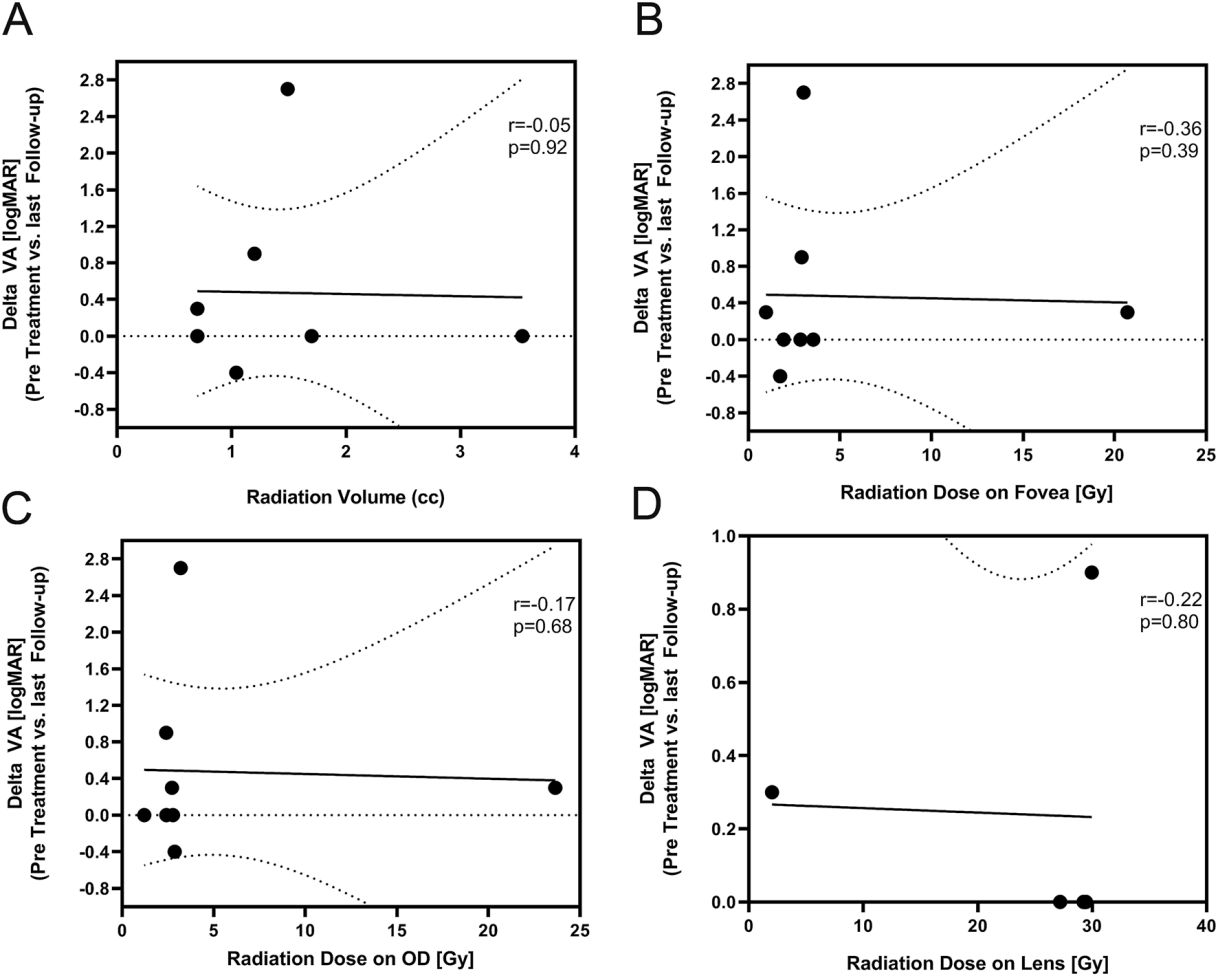
There was no correlation between total radiation dose, radiation dose on optic disc, fovea or lens with the degree of change in visual acuity.

We noticed no statistically significant impact of applied radiation volume (*p* = 0.92) or radiation dose delivered to fovea (*p* = 0.39), optic disc (*p* = 0.68) or lens (*p* = 0.80) on visual outcome at last follow-up (Fig. 3).

### ***Tumor classification according to AJCC classification (8***^***th***^*** edition)***

Tumor thickness at first presentation was 2.1 mm (range: 1.0–3.6 mm) on average with the largest diameter at a mean of 5.0 mm (range: 1.9–8.3 mm). The tumor thickness was reduced at last follow-up to a mean of 1.4 mm (range: 0.50–2.5 mm) and the diameter decreased to a mean of 4.4 mm (range: 2.8–6.8 mm). The mean reduction was 0.74 mm (range: 0.50–1.10 mm) for thickness and 0.58 mm (range: 0.90–1.50 mm) regarding diameter. The reduction in tumor thickness was statistically significant (*p* = 0.01) while the change in largest basal diameter was not (*p* = 0.16).

4 patients were classified as T1 tumors (one T1a, three T1b) at first presentation whereas 4 patients were T2 tumors with ciliary body affection (three T2a, one T2c).

### Treatment modalities

**Figure 4:**
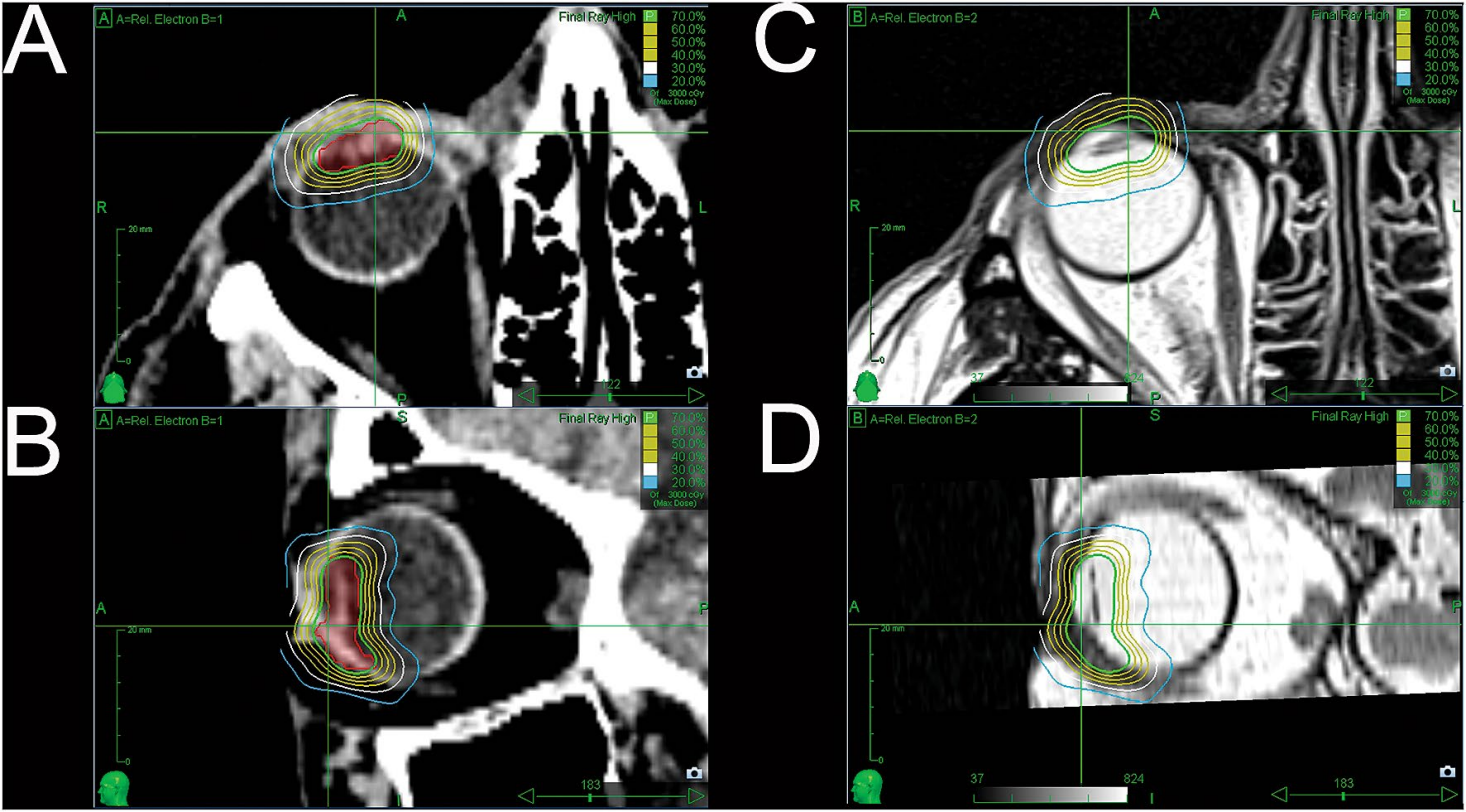
Two treatment plans are depicted in this figure. We used a donut shaped pattern in all our cases in order to make sure that the entire tumor was within the radiation field. (**A**,**B**) as well as (**C**,**D**) show the planning target volume with isodose lines from two different perspectives.

All patients were treated with 21 Gy at a 70% isodose. An inhomogeneous dose prescription is standard practice for all tumors treated with CyberKnife, and for most other radiosurgery techniques using small photon beams. An example of our treatment plan is shown in Fig. 4. Applied radiation volume was ranging from 0.70 to 3.54 mm^3^. Mean applied dosage to fovea, lens and optic disc was 4.70gy (range: 0.96–20.7), 23.58gy (range: 0.00–29.97) and 5.15gy (range: 1.20–23.65) respectively. (Table 2).

**Table 2:** Radiation treatment parameters.

	Mean (range)
Radiation dose (Gy),	21
Isodose (%)	70
Radiation volume (mm^3^)	1.4 (0.7–3.5)
Maxium radiation dose on fovea (Gy)	4.7 (1.0–20.7)
Maxium radiation dose on optic disc (Gy)	5.1 (1.2–23.7)
Treatment form	Donut

### Complication, recurrence and overall survival

Five out of eight patients (62.5%) were already pseudophakic at first presentation. Two out of three phakic patients developed cataract along the follow-up period and received cataract surgery six months and 37 months after radiation treatment.

Four patients (50.0%) developed glaucoma after a mean of 14.5 months (range 9.0–22 months) after CyberKnife treatment. Three of these patients were treated conservatively with intraocular eye pressure reducing drops, whereas one patient had glaucoma operation nine months after radiation. No patient developed neovascular glaucoma.

Radiation keratopathy was observed in five patients (62.5%). Out of these, three patients developed radiation keratopathy immediately after treatment (first follow-up visit after one month). The other two patients developed first symptoms 2 and 22 months after CyberKnife treatment. All of the patients were treated with lubricating eye drops and showed response to this treatment.

We did not notice any form of radiation retinopathy or opticoneuropathy—the central retinal thickness (CRT) showed no significant changes over the whole follow-up time.

No recurrences were appreciated over the time of follow-up and none of the patients needed enucleation for secondary complications or lack of local control. All patients remained free of metastases over the whole observation period. (Table 3).

**Table 3:** Complications following radiation treatment for iris melanoma.

	Number of patients
**Lens status**	
Phakic	5
Cataract progression	2
Surgery	2
Pseudophakic	3
**Glaucoma development**	4
Medical management	3
Surgery	1
Radiation Retinopathy	0
Radiation Keratopathy	5
Recurrences	0
Metastases	0
Enucleation	0

## Discussion

We report on 8 patients that have been treated with robotic assisted CyberKnife (Accuray, Inc.) radiosurgery due to an iris melanoma. We did not see any recurrences over the follow-up time.

Despite the rare appearance of iris melanoma and the lower rate of metastastic development with 5% at 10-year follow-up compared to otherwise located uveal melanomas^8^, iris melanoma may enlarge in size and infiltrate other tissues of the eye and may even progress to extraocular extension.

Different treatment modalities are possible and described for iris melanomas although considerably less is known on the best treatment strategy as compared to uveal melanomas in the choroid or the ciliary body. CyberKnife treatment is different compared to brachytherapy and proton beam treatment: CyberKnife comprises a linear accelerator which is mounted on an industry roboter with six degrees of freedom, allowing application of radiation from every direction. The ability of this system to deliver radiation beams from every possible angle may be advantageous when trying to save tissue from being exposed to radiation by excluding these structures during planning of treatment as much as possible. Placement of a radioactive plaques as with brachytherapy demands surgery. The plaque is directly sutured to the sclera in the region of radiation application to the underlying tissue. The time until the plaque can be surgically removed is dependent on the dose calculation of a medical physicist and radiation oncologist during treatment planning and encompasses usually a few days^9^. Proton beam therapy on the other hand is, similar to CyberKnife treatment, a teletherapeutic option in which a radiation beam is delivered in multiple fractions to the target tissue. Since the proton beam cannot be moved around the patients head, small titanium clips are sutured on the sclera before treatment in order to facilitate treatment planning and execution^17^.

Popovic et al.^18^ did a medline search of all existing treatment regimens and found a total of 17 studies with a total of 761 eyes that met their criteria for further analysis. The main treatment option for iris melanoma is brachytherapy and many reports on radiotherapy are available. Among these, brachytherapy^14,19,20^ and proton beam therapy (PBT)^13,21,22^ are the most frequently evaluated treatment approaches. Less can be found on surgical resection^18,23^.

As mentioned before, no recurrences were seen in our series of patients. These results compare well to other treatment approaches, particularly PBT or brachytherapy in which recurrence rates and metastastic development were also reported to be low. Recurrences occurred in 0–7.5% of patients following proton beam therapy^24–26^ and in 0–8%^27,28^ and up to 15%^14^ after seven years following brachytherapy. Metastases occur considerably less frequently in iris melanoma as compared to choroidal melanoma with approximately 5% after five years^10^.

Surgical resection always goes along with removal of iris tissue which subsequently increases the risk of photophobia (9–25%), yet rates of recurrences (0–8%) are not lower than for the aforementioned alternatives^29^ or the result of our study.

Due to the close proximity of the iris to the anterior chamber angle and intraocular lens, it is almost never possible to save these structures from being incorporated into the planning target volume when radiation is planned. This poses a higher risk to the development of cataract with subsequent visual acuity deterioration or secondary glaucoma caused by radiation induced structural changes in the anterior chamber angle.

Unsurprisingly, the three most commonly reported complications following PBT or plaque radiotherapy are cataract progression (36–73%), corneal discomfort and defects due to limbal cell deficiency (9–90%)^24^ and occurrence of secondary glaucoma (3–92%)^18,23^.

In all of our patients a donut shaped irradiation pattern was planned to treat the iris melanoma. This rather aggressive irradiation approach reduces the risk of missed melanoma cells in parts of the iris that are not seen clinically or with ultrasound but could possibly entail higher incidences of the aforementioned secondary complications. Notwithstanding our treatment planning, compare our results similar to published data from brachytherapy and PBT with 50% of patients developing secondary glaucoma and two out for three patients with progression of cataract and subsequent cataract surgery after 6 and 37 months. The most common finding after treatment however was keratopathy which occurred in 62.5% of all patients. This complication is often only temporary and all patients from our cohort could be managed with lubricating eye drops. Keratopathy is often not mentioned in reports on treatment outcome, so that comparison to other treatment approaches is intricate. A few reports addressing this complication exist however. Fernandes and associates^20^ for example reported mild to moderate keratitis in most of the cases (14 patients) after Idodine-125 brachytherapy for iris melanoma. In addition, Konstantinidis et al. attribute symptoms of “grittiness” and ocular discomfort observed in 67% of patients (12 patients) treated with PBT for iris melanoma to ocular surface irregularities^21^. Three out of eight patients in our study developed corneal discomfort immediately after radiotherapy and showed subjective and objective improvement (increase in visual acuity) over time. Our results regarding long-term corneal and scleral affection is in line with reports from others^20^.

Half of our patients developed glaucoma over the course of follow-up with a median occurrence after 14.5 months. Three out of four patients could be sufficiently treated with the prescription of intraocular pressure lowering eye drops. One patient needed additional glaucoma surgery to manage the increased eye pressure.

Data regarding visual acuity outcome is inhomogenous. Shields et al.^14^ reported that 37% of treated patients (52 out of 141 patients) with plaque brachytherapy would end up with poor visual acuity (< 20/200). Fernandes et al. reported increased visual acuity in one case, stability in ten cases and worsening in three cases (14 patients in total)^20^.

In our study 4 patients (50%) showed decrease in visual acuity from mean 0.3 logMAR (range: 0.10–0.70 logMAR) before treatment to a mean of 0.9 logMAR (range: 0.50–3.00logMAR) at last follow-up. Three patients (37.5%) remained stable and one patient (12.5%) had an increased visual acuity from 0.7logMAR to 0.3logMAR at last follow-up visit.

We did not find any correlation of the course of visual acuity and tumor size, neither for tumor height nor largest basal diameter (Fig. 2). Furthermore was no statistically significant correlation found between visual acuity and radiation dose on optic disc or fovea. (Fig. 3).

This report has several limitations. The retrospective character, although typical for reports on outcome of ocular cancers, does not always allow a complete record of data. In addition, is our follow-up period rather short so that statements regarding metastasis or recurrences must be interpreted with caution as iris melanoma recurrences are less common than with other uveal melanomas but may occur later in the course of observation^12^. Further, complications may also occur later in time and additional reports with a higher patient number and follow-up time are demanded to better assess this question. The small number of patients is a drawback, which does not allow general prediction on treatment outcome in linear accelerator treated iris melanoma.

However, to our knowledge there are no other reports reporting clinical outcome after robotic assisted CyberKnife treatment for iris melanoma. CyberKnife can be facilitated on one day in just over three hours including treatment planning and execution with an experienced interdisciplinary team comprising radiation oncologists, medical physicists and ophthalmologists. All of our patients showed local control after up to 48 months of post treatment observation and none of the patients had documented metastases. Complications were comparable to brachytherapy and PBT. We therefore believe that CyberKnife is a safe, effective and a comfortable option in selected cases of iris melanomas.
